# Complete Genome Sequences of Chikungunya Viruses Isolated from Plasma Specimens Collected from Haitians in 2014

**DOI:** 10.1128/genomeA.00148-17

**Published:** 2017-04-13

**Authors:** Sarah K. White, J. Glenn Morris, Maha A. Elbadry, Valery Madsen Beau De Rochars, Bernard A. Okech, John A. Lednicky

**Affiliations:** a Department of Environmental and Global Health, College of Public Health and Health Professions, University of Florida, Gainesville, Florida, USA; b Emerging Pathogens Institute, University of Florida, Gainesville, Florida, USA; c Department of Medicine, College of Medicine, University of Florida, Gainesville, Florida, USA; d Department of Health Services Research, Management and Policy, University of Florida, Gainesville, Florida, USA

## Abstract

Ten chikungunya virus isolates from human plasma collected in Haiti from May to August 2014, in the midst of a chikungunya fever outbreak, were fully sequenced. The resulting genomic sequences are nearly identical, and phylogenetic analyses indicate they belong to the Asian lineage of the virus.

## GENOME ANNOUNCEMENT

Chikungunya virus (CHIKV) (genus *Alphavirus*, family *Togaviridae*) is a mosquito-borne virus that is the cause of chikungunya fever (CF) in humans. Although infections by CHIKV may be asymptomatic, up to 97% of those infected develop CF, which is a fever-rash-arthralgia syndrome often associated with debilitating pain that may last up to several years (1). CHIKV has been isolated in regions in Asia, Africa, and the Pacific Islands, and it emerged in the Americas for the first time in 2013 with sustained autochthonous transmission (2, 3). There is little CHIKV information from Haiti, but there is a growing body of information from other countries involved in the epidemic in the Americas and Caribbean (4).

The CHIKV genomes that were sequenced here were from viruses isolated from human plasma samples obtained from schoolchildren in Haiti during a CF outbreak, between 29 May and 13 August 2014. Plasma samples from suspected cases of CF were screened by reverse transcription (RT)-PCR; cDNA synthesis from extracted RNA was performed using a Moloney murine leukemia virus reverse transcriptase (MMLV-RT) and sequence-specific reverse primers, and PCR was performed as described by Cherabuddi et al. (5). For sequencing purposes, virus isolation in a BSL3 laboratory was accomplished by inoculation of aliquots of plasma that tested positive for CHIKV viral RNA (vRNA) by RT-PCR onto Vero E6 cells. The viruses induced rapid formation of cell-lytic cytopathic effects, usually within 4 days after inoculation of the cells. Thereafter, the viral genomes were Sanger sequenced using a high-fidelity reverse transcriptase for cDNA synthesis, followed by PCR with a high-fidelity thermal polymerase, as well as 5′ and 3′ rapid amplification of cDNA ends (RACE) performed as previously described (5).

The 10 genome sequences are nearly identical (they share 99% identity) and also have 99% identity with CHIKV strain WHCHK9, which was also collected from Haiti in May 2014 (complete genome deposited by the CDC in GenBank, accession number KR559478). Presently, three distinct CHIKV lineages have been identified: African, East/Central/South African (ECSA), and Asian (6 – 8). Phylogenetic analyses indicate that our 10 CHIKV genomes belong to the Asian lineage of the virus. All 10 virus genomes harbor an E1-A98T substitution and lack an E1-A226V substitution (they retain A at E1-226). This finding suggests they are mainly vectored by *Aedes aegyptii* (7, 9, 10). In support of that notion, the 10 CHIKV genomes also lack E1 and E2 *Aedes albopictus*-adaptive mutations that are seen in IOL strains (11), but this remains to be confirmed by field studies in Haiti.

### Accession number(s).

The 10 complete CHIKV genome sequences of this study have been deposited in the GenBank database under the accession numbers KX702401, KX702402, and KY415978 to KY415985.

## References

[1] Weaver SC, Lecuit M 2015 Chikungunya virus and the global spread of a mosquito-borne disease. N Engl J Med 372:1231–1239. doi:10.1056/NEJMra1406035. 25806915

[2] Stapleford KA, Moratorio G, Henningsson R, Chen R, Matheus S, Enfissi A, Weissglas-Volkov D, Isakov O, Blanc H, Mounce BC, Dupont-Rouzeyrol M, Shomron N, Weaver S, Fontes M, Rousset D, Vignuzzi M 2016 Whole-genome sequencing analysis from the chikungunya virus Caribbean outbreak reveals novel evolutionary genomic elements. PLoS Negl Trop Dis 10:e0004402. doi:10.1371/journal.pntd.0004402. 26807575 PMC4726740

[3] Vasconcelos PF, Calisher CH 2016 Emergence of human arboviral diseases in the Americas, 2000–2016. Vector Borne Zoonotic Dis 16:295–301. doi:10.1089/vbz.2016.1952. 26991057

[4] Pan American Health Organization, WHO 2016 Number of reported cases of chikungunya fever in the Americas, by country or territory, 2016 EW 27. WHO, Geneva, Switzerland.

[5] Cherabuddi K, Iovine NM, Shah K, White SK, Paisie T, Salemi M, Morris JG Jr, Lednicky JA 2016 Zika and chikungunya virus co-infection in a traveller returning from Colombia, 2016: virus isolation and genetic analysis. JMM Case Rep 2016:3. doi:10.1099/jmmcr.0.005072. PMC5343122 28348794

[6] Powers AM, Brault AC, Tesh RB, Weaver SC 2000 Reemergence of chikungunya and O’nyong-nyong viruses: evidence for distinct geographical lineages and distant evolutionary relationships. J Gen Virol 81:471–479. doi:10.1099/0022-1317-81-2-471. 10644846

[7] Tsetsarkin KA, Chen R, Leal G, Forrester N, Higgs S, Huang J, Weaver SC 2011 Chikungunya virus emergence is constrained in Asia by lineage-specific adaptive landscapes. Proc Natl Acad Sci U S A 108:7872–7877. doi:10.1073/pnas.1018344108. 21518887 PMC3093459

[8] Weaver SC, Forrester NL 2015 Chikungunya: evolutionary history and recent epidemic spread. Antiviral Res 120:32–39. doi:10.1016/j.antiviral.2015.04.016. 25979669

[9] Weaver SC 2014 Arrival of chikungunya virus in the new world: prospects for spread and impact on public health. PLoS Negl Trop Dis 8:e2921. doi:10.1371/journal.pntd.0002921. 24967777 PMC4072586

[10] Chen R, Puri V, Fedorova N, Lin D, Hari KL, Jain R, Rodas JD, Das SR, Shabman RS, Weaver SC 2016 Comprehensive genome scale phylogenetic study provides new insights on the global expansion of chikungunya virus. J Virol 90:10600–10611. doi:10.1128/JVI.01166-16. 27654297 PMC5110187

[11] Tsetsarkin KA, Chen R, Yun R, Rossi SL, Plante KS, Guerbois M, Forrester N, Perng GC, Sreekumar E, Leal G, Huang J, Mukhopadhyay S, Weaver SC 2014 Multipeaked adaptive landscape for chikungunya virus evolution predicts continued fitness optimization in *Aedes albopictus* mosquitoes. Nat Commun 5:4084. doi:10.1038/ncomms5084. 24933611 PMC7091890

